# Effective non-surgical management of spinal cord compression from extra-medullary hematopoiesis with radiotherapy in thalassemia intermedia: a case report

**DOI:** 10.1093/omcr/omaf039

**Published:** 2025-05-28

**Authors:** Zoheir Reihanian, Nooshin Zaresharifi, Behrad Eftekhari, Anita Khalili, Ainaz Sourati, Fatemeh Nejatifar

**Affiliations:** Neuroscience Research Center, School of Medicine, Guilan University of Medical Sciences, Rasht, Guilan, Iran; Neuroscience Research Center, School of Medicine, Guilan University of Medical Sciences, Rasht, Guilan, Iran; Department of Medicine, Guilan University of Medical Sciences, Rasht, Guilan, Iran; Department of Medicine, Guilan University of Medical Sciences, Rasht, Guilan, Iran; Department of Radiation Oncology, Guilan University of Medical Sciences, Rasht, Guilan, Iran; Department of Hematology and Oncology, Guilan University of Medical Sciences, Rasht, Guilan, Iran

**Keywords:** extramedullary Hematopoiesis, spinal cord compression, beta-thalassemia, radiotherapy, case report

## Abstract

Spinal cord compression (SCC) secondary to extramedullary hematopoiesis (EMH) represents a rare but critical manifestation in patients with hematologic conditions, including β-thalassemia intermedia (β-TI). While there is no standard guideline due to the rarity of EMH-induced SCC, this case report documents successful management with radiotherapy while avoiding high-risk surgical intervention. The patient was a 17-year-old male with β-TI who developed debilitating SCC attributable to a paraspinal EMH mass. Due to concerns regarding surgical morbidity, a 26Gy 13-session course of radiation was administered alongside adjunctive blood transfusions, Hydroxyurea, and Glucocorticoids. Marked improvement and subsequent resolution of neurological deficits occurred rapidly following treatment, with sustained regression of the EMH mass over 18 months of follow-up. The case demonstrates that radiotherapy can be an effective primary treatment for EMH-induced SCC in β-TI, emphasizing its safety and efficacy when immediate and significant clinical response is required.

## Introduction

Initially characterized in 1955 by Sturgeon et al., β-thalassemia intermedia (β-TI) is a hematological disorder representing an intermediate clinical phenotype between major thalassemia and thalassemia trait [1]. Patients with thalassemia major are typically dependent on frequent blood transfusions early in life to regulate overactive bone marrow; however, this is not the case with β-TI patients, who generally do not need regular transfusions [2]. The main clinical features of β-TI stem from chronic anemia and inefficient blood cell production (ineffective erythropoiesis), leading to the enlargement of blood-forming tissues beyond the bone marrow cavity, an emergent condition referred to as extramedullary hematopoiesis (EMH) [2]. Almost all regions may be impacted by EMH, with notable prevalence in the thoracic and abdominal masses (88%), paraspinal region (39%), and involvement of the liver and spleen, while rare occurrences may be noted in the sinus cavities and other atypical sites [3, 4]. A significant complication associated with EMH in the spinal canal is spinal cord compression (SCC), which substantially hinders patient mobility and is primarily caused by oncological conditions. However, EMH may also manifest in various chronic hematological disorders, including myelofibrosis, polycythemia vera, diverse anemias, hematologic malignancies, and post-radiation scenarios [5]. The current case report focuses on an individual with β-TI who developed SCC due to EMH and subsequently underwent radiotherapy. This report intends to illustrate the positive outcomes of radiotherapy, specifically in achieving local tumor control, reducing pseudo-tumor size, and alleviating symptoms related to SCC in the context of β-TI.

## Case report

**Figure 1:**
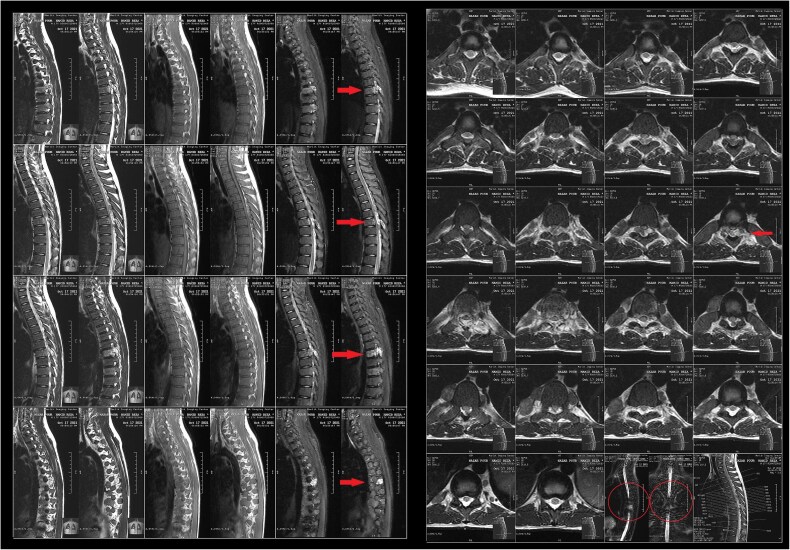
(A) Sagittal and (B) axial T2-weighted MRI images of the dorsal spine, revealing diffuse marrow signal abnormality in the lumbar region, a finding that strongly suggests chronic congenital anemia (thalassemia). Multiple ovoid variable sizes low T1 and T2 signal soft tissue lesions in the right and left of T6, T7, T8, T9, and T9-10 levels, an indicator of extra-medullary hematopoiesis (arrows). A mixed T1 and T2 signal soft tissue in the posterior epidural space at the T6-7, T7, and T8 levels is causing pressure on the thecal sac and cord, leading to canal stenosis at the mentioned levels with edema in the cord (circles), a sign of SCC due to this soft tissue.

**Figure 2:**
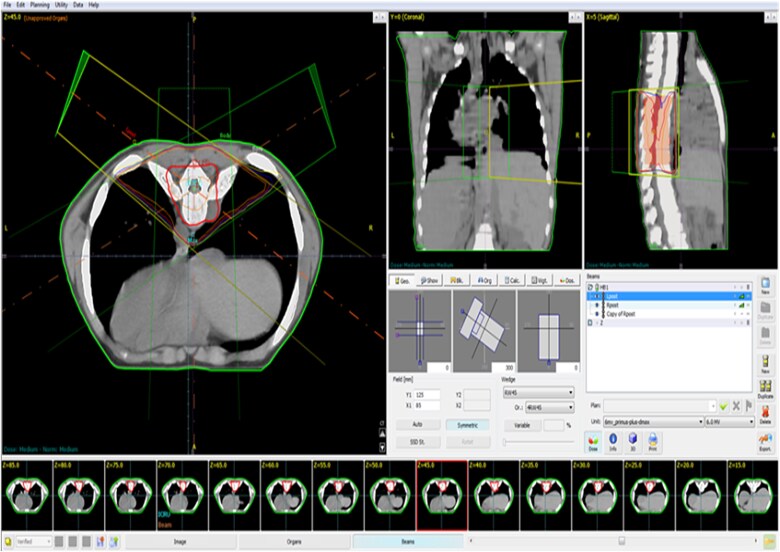
Three-dimensional conformal radiation therapy planning, iso-dose distribution.

**Figure 3:**
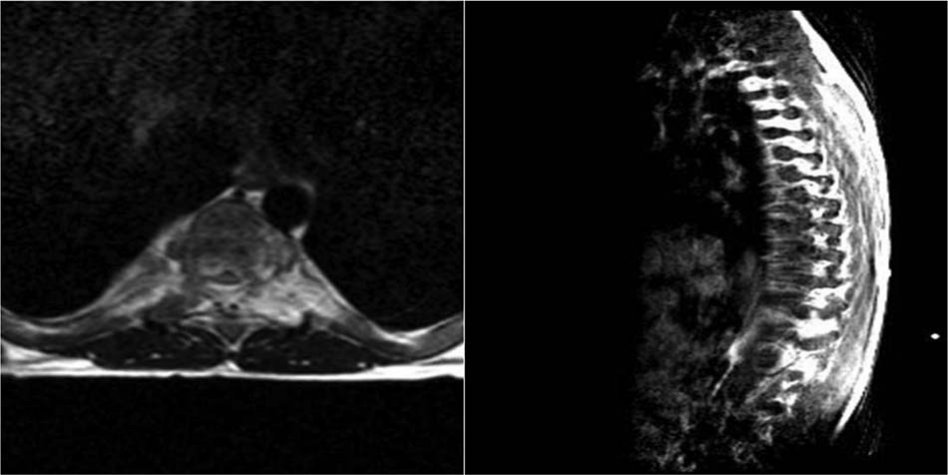
(A) Axial and (B) Para-sagittal T2W MRI of dorsal spine after radiation treatment reveals no evidence of lesion.

A 17-year-old male with a known history of β-TI presented with one month of bilateral dorsal foot pain and two weeks of progressive left lower extremity weakness. Physical examination revealed 3/5 strength on left proximal lower limb muscle testing and dorsiflexion. Signs of upper motor neuron pathology were evident, including hyperreflexia and bilateral clonus without sphincter dysfunction. Laboratory analysis indicated a hemoglobin of 8 g/dl, hematocrit of 24.2%, mean corpuscular values consistent with microcytic anemia, and adequate platelet levels (Table 1). Magnetic resonance imaging (MRI) of the thoracic spine showed a paravertebral soft tissue mass at T7-T8 (Fig. 1), causing spinal cord compression, consistent with EMH. Initial neurosurgical consultation advised against surgical intervention, given risks from mass hypervascularity and patient osteopenia. Thus, radiation oncology provided definitive management to avoid high-risk surgery. The patient underwent radiation therapy with 26Gy over 13 fractions (5 fractions a week) (Fig. 2), alongside transfusional support and Hydroxyurea (500 mg daily) for EMH suppression and Corticosteroids (Dexamethasone 8 mg, daily) to prevent spinal cord edema. Marked clinical improvement occurred within a few days of starting radiotherapy as left lower limb strength normalized (5/5). A follow-up MRI conducted 30 days post-radiation revealed a complete radiographic resolution of the EMH mass alongside successful spinal decompression (Fig. 3). Additionally, a complete blood count performed at this time indicated improvements in hemoglobin, hematocrit, and erythrocyte parameters (Table 1). Hydroxyurea was discontinued during the 6-month follow-up, with sustained improvements in hemoglobin levels noted. Throughout a 30-month period of continuous surveillance, the patient remained asymptomatic and exhibited no adverse side effects.

**Table 1:** Patient’s hematologic evaluation.

**CBC Parameters**	**Before Radiation**	**After Radiation**
**WBC** (cells/mcl)	6900	6100
**RBC** (10^6^ cells/mcl)	2.93	3.71
**Hb** (g/dl)	8	10.8
**Hct** (%)	24.2	31.3
**MCV** (fl)	82.5	84.4
**MCH** (pg)	33	30
**MCHC** (g/dl)	33	34.5
**Plt** (cells/mcl)	118 000	369 000

## Discussion

Beta-thalassemia exhibits a significant incidence in geographic areas, including the Mediterranean, Africa, and parts of the Middle East (containing Iran), with mutation frequencies ranging from 3% to 10% in specific locales [6]. In Iran, the condition predominantly appears along the coastal regions of the Persian Gulf and the Caspian Sea, where it surpasses a 10% prevalence rate [6]; the setting for our case also falls within this area. EMH manifests as a frequent complication among patients with intermediate or major thalassemia requiring regular transfusions, acting as a physiological response to ineffective erythropoiesis with its diagnosis being typically linked to chronic hemoglobinopathies coupled with an MRI representing an epidural soft-tissue mass [5]. SCC, while not a common consequence of EMH in thalassemia patients, is remarkable due to its potential to induce severe and possibly irreversible neurological impairments, and the most effective way to manage this complication is still controversial, as no clear evidence exists to show which treatment option is optimal [7]. This is due to the rarity of the disease, the absence of randomized clinical trials, and the uncommon incidence of symptomatic cord compression cases [7]. Although hypertransfusion strategies might diminish the need for extramedullary hematopoiesis, side effects remain a concern, and there have been reported instances where it did not prevent spinal cord compression, which was subsequently resolved by radiotherapy [8]. Hydroxyurea, known to boost fetal hemoglobin levels, has been mentioned for reducing SCC in case studies, yet it typically requires several weeks to take effect and may still necessitate radiation upon relapse [9]. In this report, we highlight the case of a 17-year-old male with beta-thalassemia intermedia who experienced progressive pain in his dorsal foot and weakness in the lower limbs. He underwent low-dose radiation treatment that fell within spinal cord tolerance levels and exhibited significant symptomatic improvement without any noted complications. With numerous studies demonstrating its effectiveness in resolving neurological symptoms and providing significant radiological resolution, radiation therapy has become the favored treatment option [10]. Our findings align with documented cases where patients with thalassemia-related SCC due to EMH underwent radiotherapy and emerged with markedly improved neurological functions. It is worth mentioning that while radiation therapy offers a low-risk and generally well-tolerated treatment method, concerns have been raised regarding the loss of tissue for pathological examination post-treatment. Modern diagnostic techniques, particularly MRI, mitigate this issue, reserving tissue biopsy for cases with high malignancy suspicion or indeterminate diagnoses due to the massive hemorrhage risk [11]. Radiation therapy may come with downsides, such as a potential decline in hematopoiesis, exacerbating anemia, and transient neurologic setbacks due to radiotherapy-induced swelling; these effects, however, can be managed with additional treatments [12]. Routine blood count monitoring and blood transfusions, as well as steroid medications, can prevent adverse symptoms during radiation therapy sessions. Furthermore, the potential for EMH recurrence following only radiation therapy is documented in a range of 19% to 37% of cases [8] and can often be effectively managed with additional radiation doses. A systematic review by Lam et al. identified two relapse cases among the 54 patients analyzed; both occurred in patients who had experienced delayed initial treatment (3 months and 6 years from symptom onset, respectively) [13]. In this case, the patient underwent both medical and radiation therapies and sustained a symptom-free status with no clinical or radiological indications of recurrence over a follow-up duration of 30 months. Notably, the patient’s hemoglobin levels remained stable throughout this timeframe, contributing to the favorable outcome while decreasing the likelihood of recurrence. To conclude, our stance advocates using radiotherapy as the primary treatment for SCC resulting from EMH, given its capacity for quick symptom resolution, sustained effect, and limited acute hematologic and gastrointestinal side effects. To substantiate this evidence, it is essential to advocate for the initiation of further clinical research in the near future.

## Consent

The study was published with the written consent of the patient’s parents/legal guardian.

## Guarantor

Ainaz Sourati M.D. (corresponding author).
